# PURE PRIME: Implementing PUlmonary REhabilitation in PRIMary carE: a protocol for a randomized controlled feasibility trial

**DOI:** 10.1017/S1463423625100340

**Published:** 2025-08-15

**Authors:** Jessica A. Walsh, Zoe J. McKeough, Marita T. Dale, Jennifer A. Alison, Sarah M. Dennis

**Affiliations:** 1 Sydney School of Health Sciences, Faculty of Medicine and Health, The University of Sydney, Camperdown, NSW, Australia; 2 Allied Health, Sydney Local Health District, Camperdown, NSW, Australia; 3 South Western Sydney Local Health District, Liverpool, NSW, Australia; 4 Ingham Institute of Applied Medical Research, Liverpool, NSW, Australia

**Keywords:** Chronic obstructive pulmonary disease, Interstitial lung disease, Primary care, rehabilitation

## Abstract

**Introduction:**

The benefits of pulmonary rehabilitation (PR) on exercise capacity, health-related quality of life (HRQoL), and prevention of readmission post exacerbation in chronic respiratory diseases (CRD) are well established. However, accessibility to PR programmes is limited by PR programmes mostly being available through hospital clinics only. Utilizing existing workforce and infrastructure in private physiotherapy and exercise physiology practices may be a solution to increase access.

**Methods:**

A mixed-methods assessor-blinded randomized controlled feasibility trial will be conducted in two parts. First, the efficacy of a training programme for private practice (PP) physiotherapists and accredited exercise physiologists who have not previously provided PR will be evaluated. Participant knowledge, skills, and confidence to provide PR will be measured before and after the training and at three months follow-up. Secondly, patient participants with CRD will be randomly allocated to receive twice weekly PP PR for 8 weeks or usual care from their general practitioner (GP). Exercise capacity, HRQoL, and health status will be measured before and after PR. A purposive sample of clinician and patient participants will partake in semi-structured interviews at the study conclusion. Interviews will continue until data saturation is achieved.

**Discussion:**

This study will provide data on the feasibility of providing PR by physiotherapists and exercise physiologists in the PP setting. Provision of PR in the PP setting has the potential to increase access to this highly evidence-based intervention to improve outcomes for people with CRD.

## Introduction

### Background and rationale

Chronic respiratory diseases (CRD) are one of the leading causes of morbidity and mortality worldwide and account for a substantial healthcare expenditure (Vos *et al.*, 2020). Pulmonary rehabilitation (PR) is an exercise and education programme for people with CRDs that improves exercise capacity and health-related quality of life (HRQoL) (McCarthy *et al.*, 2015), as well as reducing hospital readmissions (Moore *et al.*, 2016). PR is a cost-effective intervention with a dominant incremental cost per quality-adjusted life year (Deloitte Access Economics, 2018). National and international guidelines recommend people with CRDs should undertake PR (Bolton *et al.*, 2013, Alison *et al.*, 2017, Rochester *et al.*, 2023).

Despite the effectiveness of PR, as little as 5–10% of eligible patients have access to a PR programme (NSW Agency for Clinical Innovation, 2010). Fewer still complete the programme and, therefore, do not achieve full benefits (Singh *et al.*, 2020). Accessibility to programmes is a key barrier to patients undertaking and completing PR. Accessibility encompasses issues around location, travel, timing of programmes, and waiting list times (Johnston *et al.*, 2011, Cox *et al.*, 2017). Alternative models of PR that overcome these barriers have been flagged as a research priority (Rochester *et al.*, 2015). There has been an emergence of alternative models of PR that aim to address barriers to PR, including telerehabilitation (Cox *et al.*, 2021) and home-based PR (Holland *et al.*, 2017). However, these two models involve either unsupervised or remote exercise sessions and are largely staffed by existing PR services staff. Models of PR that are provided outside of existing secondary care services are needed to expand the available trained workforce. Environmental factors were the most frequently referenced domain affecting PR referral, uptake, completion, and attendance, and these factors represent 37% of mapped barriers or enablers (Cox *et al.*, 2017).

Globally, most people with CRDs are managed in the primary care setting by their general practitioner (GP) (Levy *et al.*, 2006). Conversely, PR programmes are primarily conducted in hospital outpatient settings (Johnston *et al.*, 2011, Singh, 2020). These programmes operate with long waitlists and there are environmental barriers related to transport, parking, and accessibility for people with respiratory conditions. PR conducted in community settings has been shown to have similar efficacy to hospital-based programmes (McNamara *et al.*, 2016). However, in practice these programmes are often still staffed by hospital employed health professionals.

There is an untapped existing primary care workforce and infrastructure of private practice (PP) physiotherapists and accredited exercise physiologists (AEPs), professions that are accredited to provide PR in primary care. For example, Australian health workforce data show that only 28% of physiotherapists and 7% of AEPs, the key professions that deliver PR, work in the hospital sector (Department of Health, 2019, Exercise & Sports Science Australia, 2023). However, 93% of Australian PR programmes are provided by hospital employed staff (Johnston *et al.*, 2011) and in the UK, 94% of programmes are provided by public organizations (Singh, 2020). The majority of these allied health professionals work outside of the hospital sector, with 57% of Australian AEPs and 40% of physiotherapists working in PPs (Exercise & Sports Science Australia, 2015, Australian Physiotherapy Association, 2023). Due to the sheer number of private practitioners and practices, private clinics close to patients’ homes may increase accessibility of PR.

Despite a large workforce credentialed to deliver PR, there is currently no funding model to support PR in the PP setting in most countries. Recent position statements identify the gap in utilization of PP allied health professionals in the management of chronic disease, and call for an expansion of the funding to allow for physiotherapists and AEPs to deliver PR wholly within the PP setting (Exercise & Sports Science Australia, 2021, Lung Foundation Australia, 2022). There are multiple reasons for this gap, including funding, training, and facilities. Currently, private physiotherapists and AEPs in Australia operate on a fee for service model. There are no subsidies to fund a complete PR programme, meaning patients would need to have private health insurance or pay fees out-of-pockets. There is a need for evidence of feasibility and effectiveness of PR delivered in the PP setting to support calls for a policy change in funding.

To date, trials implementing PR in PP have not provided an intervention consistent with international PR guidelines (Bolton *et al.*, 2013, Alison *et al.*, 2017, Rochester *et al.*, 2023). These guidelines recommend that PR programmes be at least eight weeks duration, with a minimum of two supervised exercise sessions per week and one unsupervised session (Alison *et al.*, 2017). Casey *et al.* (2013) conducted a large randomized controlled trial (RCT) comparing once weekly PR in primary care to usual care of no PR. The study showed a statistically but not clinically significant between-group improvement in HRQoL in favour of the PR group but no difference in exercise capacity (Casey *et al.*, 2013). The reduced frequency of PR in the intervention compared to national (Alison *et al.*, 2017) and international guidelines (Bolton *et al.*, 2013) may have contributed to this result. There have been smaller studies conducted, however, they also failed to meet guidelines (Zakrisson *et al.*, 2011, Anastasaki *et al.*, 2019) or were not conducted solely in primary care (Cecins *et al.*, 2017). It is also important to understand the upskilling required for physiotherapists and exercise physiologists to provide PR in primary care. In a systematic review on educational interventions for healthcare professionals for chronic obstructive pulmonary disease (COPD) management in primary care, only one RCT in this review evaluated education for physiotherapists or exercise physiologists providing COPD management, with no specific education on the provision of PR (Cross *et al.*, 2022), indicating a gap in the literature about upskilling physiotherapists and AEPs in this area of practice.

The current study seeks to bridge these knowledge gaps and examine whether it is feasible to provide an evidence-based PR programme provided by physiotherapist or AEPs in their PPs. It is also vital to gather data for the Australian-specific context. There is a critical need to determine how PR can be implemented more widely for people with CRD so that they benefit from the well-established and multidimensional improvements.

## Objectives

Part One: The primary objective is to investigate the efficacy of a PR training programme for PP physiotherapists and AEPs to develop the knowledge, skills, and confidence necessary to conduct a PR programme. The secondary objective is to identify if the training programme meets the needs of the PP clinician participants and is acceptable to them.

Part Two: The primary objective is to investigate whether it is feasible for PP physiotherapists and AEPs to conduct PR in primary care. Secondary objectives are: (i) to assess, in participants with CRD whether PR conducted in primary care improves exercise capacity, HRQoL, and health status compared to usual medical care provided by a GP; (ii) to investigate whether the delivery of PR is acceptable from the perspective of PP physiotherapists and AEPs; and (iii) to provide data to allow for the design of a full-scale definitive trial.

We hypothesize that after appropriate upskilling of clinicians, PR provided in primary care will be acceptable to PP clinicians and patients, feasible to implement, and will be associated with increased exercise capacity and HRQoL in people with CRD when compared to usual care.

## Methods and analysis

### Participants, interventions, and outcomes

#### Trial design

The study will be a mixed-methods assessor-blinded randomized controlled feasibility trial, conducted in two phases. Part One will involve training clinician participants in PR and Part Two will involve a randomized controlled trial of PR delivered to patient participants by those clinicians upskilled in Part One compared to usual care.

#### Participant timeline

**Figure 1.**
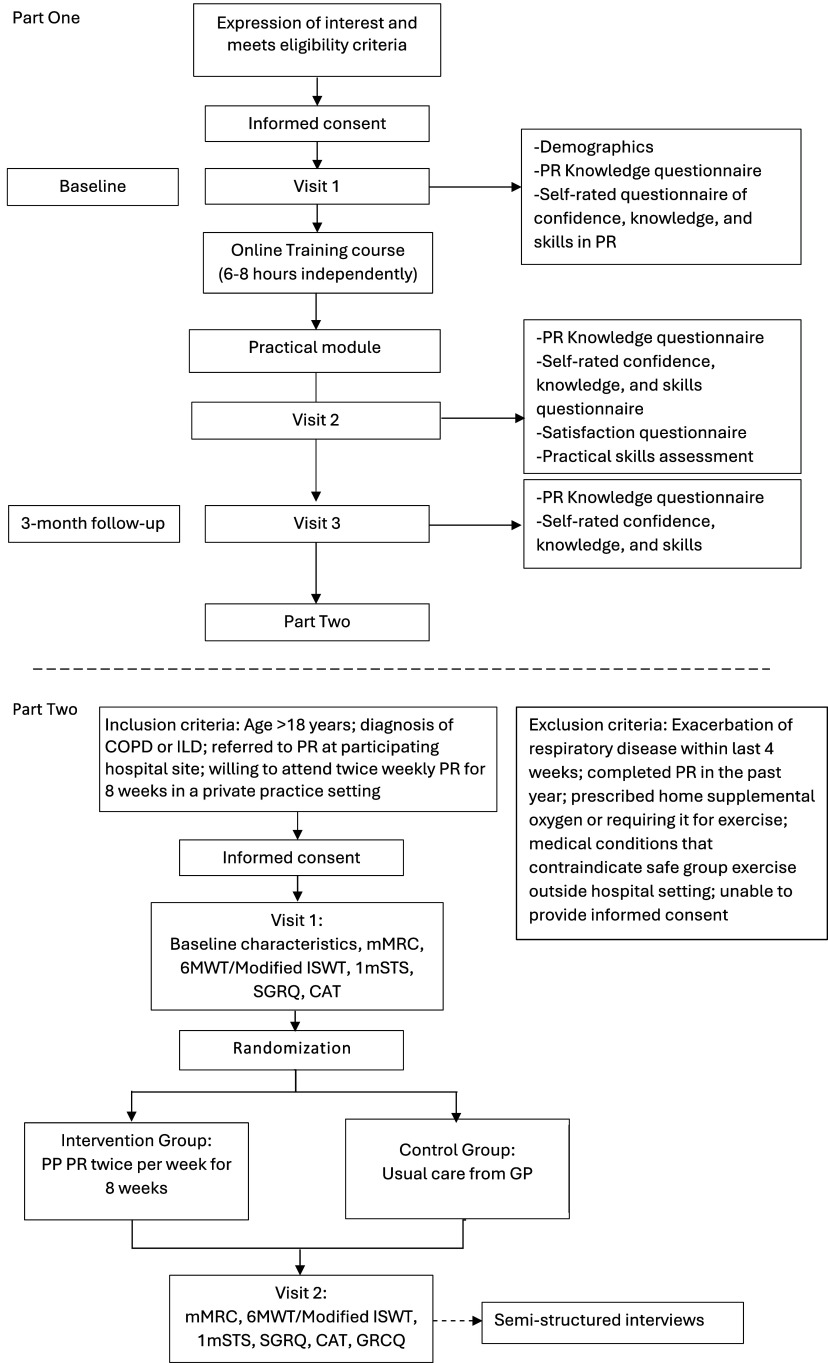
Part One and Two participant timelines. Abbreviations: COPD, chronic obstructive pulmonary disease; ILD, interstitial lung disease; OPD, outpatient department; PR, pulmonary rehabilitation; mMRC, modified Medical Research Council dyspnoea scale; 6MWT, six-minute walk test; ISWT, incremental shuttle walk test; 1mSTS, 1-minute sit-to-stand; SGRQ, St George Respiratory Questionnaire; CAT, COPD Assessment Test; PP, private practice; GP, General Practitioner; GRCQ, Global Rating of Change Questionnaire.

A timeline for participants in both parts of the study is outlined in Figure 1.

#### Study setting

The study will be conducted within the geographical area of three local health districts (LHDs) in metropolitan Sydney, Australia. Patient participant assessments and intervention will be conducted wholly within the participating physiotherapy or AEP practices.

#### Participants

Clinician participants will be recruited for Part One and patient participants recruited for Part Two. The inclusion and exclusion criteria for both participant groups are outlined in Table 1.

**Table 1.** Inclusion and exclusion criteria for clinician and patient participant groups

	Clinician participants	Patient participants
Inclusion criteria	- Registered physiotherapist or accredited exercise physiologist - Working in private practice where: o At least two staff (clinical or administrative) are present during assessments and group exercise sessions o There is adequate space for group exercise for a minimum 3 participants o There is at least 10m straight distance to perform a field walking test o Accessible for people with chronic respiratory disease - Willing to provide intervention for part two of study (twice weekly PR for 8 weeks) - Have or be willing to obtain current CPR training prior to assessing any study participants	- Age >18 years - Diagnosis of COPD or ILD (confirmed with spirometry (Williams *et al.,* 2014) or diagnosis by medical doctor) - Referred to pulmonary rehabilitation at one of three metropolitan LHD hospitals and on the waitlist - Willing to attend twice weekly PR for 8 weeks in a private practice setting
Exclusion criteria	- Physiotherapists or AEPs with significant prior PR experience (e.g. more than one rotation as Level 1 physiotherapist or AEP in a hospital PR setting or delivery of Lungs in Action) - Physiotherapists or AEPs with postgraduate cardio-pulmonary qualifications	- Exacerbation of respiratory disease within 4 weeks prior to recruitment - Completed PR in the past year - Prescribed home supplemental oxygen or requiring supplemental oxygen for exercise (desaturates < 80% SpO_2_ on field walking test) - Medical conditions that contraindicate safe group exercise outside hospital setting (e.g. unstable cardiac disease, severe musculoskeletal impairments) - Unable to provide informed consent

Abbreviations: PR, pulmonary rehabilitation; CPR, cardio-pulmonary resuscitation; AEP, accredited exercise physiologist; COPD, chronic obstructive pulmonary disease; ILD, interstitial lung disease; LHD, local health district; SpO_2_, oxygen saturation measured by pulse oximetry.

### Recruitment

Clinician participants will be recruited from study advertisements through primary health network newsletters, professional societies, and contact with PR services. Patient participants will be recruited from waitlists of existing PR services within the participating LHDs. Potential participants will be screened for eligibility from existing service waitlists by a member of the treating clinical team. Eligible participants will be contacted by the researcher.

#### Randomization, allocation, and blinding procedures

Patient participants will be randomized 1:1 to either receive the intervention or usual care. Randomization will be conducted within the randomization module of REDCap and be performed by an unblinded member of the research team. Randomization will be stratified by exercise capacity (6-minute walk distance (6MWD) ≥350m or <350m, ISWT ≥250m or <250m), and level of breathlessness with activity (modified Medical Research Council dyspnoea scale (mMRC) ≥3 or <3).

For Visit 1, clinical outcome measures will be completed by PP physiotherapists or AEPs. Randomization will occur after completion of Visit 1 data collection. The study coordinator will remain blinded to group allocation throughout the trial and will perform assessments at Visit 2. Due to the nature of the intervention, both clinician and patient participants will not be blinded to group allocation. Data will be analysed without knowledge of group allocation.

#### Interventions

##### Part one

All clinician participants will be given access to the Lung Foundation Australia Pulmonary Rehabilitation Online Training Program (https://lungfoundation.com.au/events/pulmonary-rehabilitation-online-training/). This is a 6-8 hour self-directed online training programme that increases the knowledge and skills of health professionals to deliver evidence-based PR. It has not been developed for specific professions so the same programme will be completed by physiotherapists and AEPs. Modules included are: Establishing a PR programme; The respiratory system and COPD; Comprehensive patient assessment; Spirometry; Assessing exercise capacity; Assessing quality of life; Exercise training; Patient education; Program evaluation and troubleshooting.

After completion of the online training, participants will attend a practical in-person module. The practical module will be a group session, delivered by experts in PR. The practical module will include performing field tests for measurement of exercise capacity and developing exercise prescriptions. Participants will be required to pass a predefined competency threshold. The minimum threshold for the knowledge questionnaire will be 80% of questions correct. The minimum threshold for the practical skills assessment will be achieving a rating of ‘proficient’ in three of the four components for each exercise capacity test and being proficient in the core competencies of each test.

##### Part two

Patient participants allocated to the intervention group will undertake twice weekly PR at their allocated PP for 8 weeks. PP PR programmes will have a maximum of eight participants in any session. PR sessions will follow the Australian and New Zealand PR guidelines (Alison *et al.*, 2017) and consist of at a minimum 30 minutes of lower limb endurance training (e.g., treadmill walking, ground walking, and stationary cycling), strength training and a home exercise programme. As per the training clinician participants received in Part One of the study, participants with CRD will be prescribed an exercise programme from their baseline exercise capacity assessment or a symptom-based prescription from the Borg scale for rating breathlessness or fatigue in accordance with the current evidence-based practice guidelines (Alison *et al.*, 2017). The specific exercises prescribed for each participant will be left to the discretion of the physiotherapist or AEP and will take into account available exercise equipment at their practice, participant goals, and past medical history. Clinicians will document the mode, duration, intensity, type, and progression for each exercise prescribed. A home exercise programme will also be prescribed for each participant. Intervention session logs and home exercise programme logs will be completed to document what was achieved by each participant compared to what was prescribed. This will be used to measure intervention fidelity and adherence. A sample intervention session is outlined in Table 2.

**Table 2.** Sample intervention session

Training type	Mode	Intensity	Type	Duration
Lower limb endurance (must include walking)	Walking	80% average speed on 6MWT or 75% peak speed of modified ISWT, aiming for dyspnoea or leg fatigue 3-4 (moderate to somewhat severe) on 0–10 category ratio scale (Zakrisson *et al.,* 2011)	Continuous or interval	Total 30 minutes
	Treadmill	80% average speed on 6MWT OR 75% peak speed of modified ISWT aiming for dyspnoea or leg fatigue 3–4 (moderate-somewhat severe) on 0–10 category ratio scale (Zakrisson *et al.,* 2011) . Start at speed 0.5-1km/hr lower than calculated until familiar with treadmill	Continuous or interval	
	Stationary cycle (if available)	60% peak work rate estimated from 6MWT or dyspnoea or leg fatigue of 3-4 (moderate-somewhat severe) on 0–10 category ratio scale (Zakrisson *et al.,* 2011).	Continuous or interval	
Upper and lower limb strengthening	Resistance bands or dumbbells	3 sets 8–10 repetitions for each exercise at a weight or resistance where these repetitions can just be completed		10–15 minutes
Upper limb endurance	Resistance bands or dumbbells	1-2 sets of 15–20 repetitions for each exercise, at a weight of resistance that causes muscle ‘fatigue’. Low weight, high repetitions		10 minutes

Abbreviations: 6MWT, 6-minute walk test; ISWT, incremental shuttle walk test.

The control group will receive usual care from their GP. At study completion, all participants will be offered the opportunity to return to the centre-based PR waitlist from which they were recruited.

#### Outcomes

Outcome measures for Part One and Two of the study are outlined in Table 3.

**Table 3.** Study outcome measures

Part One	Part Two
**Primary outcome**	
Change in participant knowledge as measured by knowledge questionnaire and change in participant self-rated confidence, knowledge, and skills questionnaire before and after training course.	Adherence to the intervention, measured by the proportion of randomized participants who complete the intervention. Defined as at least 12 of the possible 16 sessions (75%).
**Secondary outcomes**	
The proportion of participants meeting proficiency threshold in practical skills assessment.	The proportion of eligible participants randomized (feasibility).
Change in participant self-rated confidence, knowledge, and skills questionnaire at 3 months post training.	The proportion of PR sessions where physiotherapists or AEPs provide exercise prescriptions of appropriate intensity and duration (fidelity).
Maintenance of participant knowledge at 3-months post training course.	Change in exercise capacity (6-minute walk test or incremental shuttle walk test, 1-minute sit-to-stand test) prior to and after 8-week PP PR intervention or 8-week control period.
The proportion of participants meeting proficiency threshold in the knowledge questionnaire before, after training and at 3-months post training.	Change in HRQoL (St George’s Respiratory Questionnaire) prior to and after 8-week PP PR intervention or 8-week control period.
Change in participant self-rated confidence, knowledge, and skills questionnaire at 3-months post training.	Change in Health status (COPD Assessment Test) prior to and after 8-week PP PR intervention or 8-week control period.
Proportion of participants who go on to deliver the intervention.	Number of adverse events.

Abbreviations: PP, private practice; PR, pulmonary rehabilitation; AEP, accredited exercise physiologist; HRQoL, health-related quality of life; COPD, chronic obstructive pulmonary disease.

##### Part one

The primary outcome is change in participant knowledge as measured by knowledge questionnaire and change in participant self-rated confidence, knowledge, and skills questionnaire before and after training course. The knowledge questionnaire is an 11-item multiple choice questionnaire based on a case vignette constructively aligned to the content of the training. The questionnaire can be found in supplemental file 1. The self-rated confidence, knowledge, and skills questionnaire is a 21-item questionnaire consisting of 5-point Likert Scales.

Secondary outcomes include: the proportion of participants meeting proficiency threshold in practical skills assessment; maintenance of participant knowledge 3 months post training course; the proportion of participants meeting proficiency threshold in the knowledge questionnaire before, after training, and 3 months post training; change in participant self-rated confidence, knowledge, and skills questionnaire at 3 months post training, proportion of participants who go on to deliver the intervention.

### Part two

The primary outcome is adherence to the intervention, measured by the proportion of randomized participants who complete the intervention. Completion will be defined as attending 12 of the 16 (75%) allocated sessions (Williams *et al.*, 2014). Secondary outcomes include: feasibility – the proportion of eligible participants randomized; fidelity – the proportion of PR sessions where physiotherapists or AEPs provide exercise prescriptions of appropriate intensity and duration. The proportion of missing data will also be analysed.

Clinical outcome measures will test effectiveness. Dyspnoea will be assessed by the mMRC Dyspnoea Scale (Mahler and Wells, 1988). Exercise capacity will be measured by a field walking test and the 1-minute sit-to-stand. The field walking test performed will be the 6-minute walk test (6MWT) if there is a 30m walking track for the test to be performed according to the European Respiratory Society/American Thoracic Society (ERS/ATS) technical standards (Holland *et al.*, 2014). If there is not adequate space, the incremental shuttle walk test (ISWT) will be performed, again according to ERS/ATS technical standards. The same test will be performed throughout the study for each study participant. Both the 6MWT and ISWT have demonstrated reliability and validity and are responsive to PR (Singh *et al.*, 2014). HRQoL will be measured by the St George’s Respiratory Questionnaire (Jones *et al.*, 1991). Health status will be measured by the COPD Assessment Test (Jones *et al.*, 2009). Both questionnaires have excellent reliability and validity and are responsive to PR (Singh *et al.*, 2001, Kon *et al.*, 2013). Perception of change post intervention will be measured by a 5-point Global Rating of Change Questionnaire.

Number of adverse events will be recorded as a safety outcome measure. Adverse events will be defined as any untoward medical occurrence experienced by a study participant and that does not necessarily have a causal relationship with this treatment. Exacerbations of the participant’s CRD will not be classed as adverse events as they are an expected events due to the nature of the disease but will be recorded and reported as a separate outcome. Qualitative outcome measures will include patient participant experience of participating in the trial, clinician participant experience of participating in the trial, and barriers and facilitators to trial participation and completion.

#### Study procedures

Data for Part One of the study will be collected online by REDCap using surveys. Questionnaires used in Part One have been designed specifically for the study. Data for Part Two will be collected in person. Any participant who desaturates to SpO_2_ <80% on the field walking test will be withdrawn from the study prior to randomization.

Semi-structured interviews will be conducted with a purposeful sample in both clinician and patient participants. The interviews will explore qualitative aspects of the feasibility of the outcome measures and participant’s experience in the study. Clinician interview guides will also explore participant’s experience of upskilling in PR delivery, identifying any barriers or facilitators, and any improvements that could be made. Topic guides will also include exploration of business models and whether clinicians view the intervention as feasible from a business perspective. After the first 2 to 3 interviews, interview guides will be revised if necessary.

The interviews will take place after the completion of Part Two of the study (or earlier if there is attrition). A purposive sampling of patient participants will be taken to capture those who complete or do not complete the intervention. Interviews will be conducted by members of the study team by videoconferencing or phone. Interviews will be audio recorded with consent and transcribed verbatim. Transcripts will be offered to the participant to check for accuracy.

### Statistical methods

#### Sample size

Fifteen clinician participants will be recruited for Part One to detect a 7.6-point change in participant knowledge postupskilling with 80% power and a standard deviation of 4.3 points, accounting for 20% attrition. This sample size is informed by a previous study that utilized a similar questionnaire (Johnston *et al.*, 2013). For Part Two, as this is a feasibility trial to inform a future definitive trial, a formal sample size calculation is not appropriate. However, Browne et al. (Browne, 1995) recommend a minimum of 30 participants per arm to estimate a parameter for any future sample size calculation. Therefore, allowing for 20% attrition rate, a total of 76 participants will be recruited.

#### Quantitative data analysis

Quantitative data will be analysed using SPSS statistics package.

##### Part one

Quantitative data will be first summarized using descriptive statistics. Likert scales will be analysed as ordinal scales, 5-point scales will have values from -2 to +2 assigned. Change data will be analysed over all timepoints using ANOVA, followed by t-tests to determine where significant change occurred. Post-hoc type I error control measures will be employed. Proportions of participants meeting the defined levels of competency for the practical skills assessment will be calculated.

##### Part two

Data will be analysed in accordance with CONSORT extension for randomized pilot and feasibility trials guidelines. Intention-to-treat analysis will be used. Feasibility outcomes will be described using proportions and corresponding 95% confidence intervals (CI), or for continuous variables means, standard deviations, and ranges. The proportion of missing data will be summarized for each clinical outcome at each time point. Where possible, reasons for missing data will be recorded. Descriptive statistics will summarize clinical outcomes and effect sizes estimated. Completion rates, missing data, variances, difference, and 95% CIs pre- and post-intervention as well as between-group differences will be calculated. Change data for field walking tests will be presented as percentage changes. The proportion of participants who meet the minimum clinically important differences for each test will be calculated. For all other clinical outcomes, change data will be presented as absolute changes.

#### Qualitative data analysis

Semi-structured interviews will be transcribed verbatim. Interview transcripts will be examined for emergent themes using an inductive thematic analysis by six key stages. These stages being familiarization, coding, searching for themes, reviewing themes, defining themes, and reporting (Braun and Clarke, 2006). The Theoretical Framework of Acceptability will be used to inform these themes (Sekhon *et al.*, 2017). NVivo will be used to assist analysis. Recruitment for interviews will continue until data saturation is achieved. This will be defined as no new themes being identified in three consecutive interviews.

### Data management and monitoring

Study data will be stored on secure REDCap databases, housed within the University of Sydney servers. A full audit trail of data entry and changes is automatically recorded by REDCap. Data files will be stored securely on the University’s Research Data Store. All adverse events will be reported through local ethics committee and Sponsor pathways. Any participant who is withdrawn after randomization for safety reasons will not be replaced. An independent 10% quality check will be performed for each study visit for the two trial parts. If more than 10% error from source data is identified, further validation will be performed using source data.

### Study timelines

Participant recruitment for Part One will occur from March 2023 to March 2024, with data collection expected to be completed in June 2024. Recruitment for Part Two of the study is expected to run from September 2023 to July 2025, with data collection completed in September 2025.

### Ethics and dissemination

This study has received approval from Sydney Local Health District (RPAH Zone) Human Research Ethics Committee (2022/ETH02324). It has been prospectively registered with the Australian and New Zealand Clinical Trials Registry (ACTRN12622001501730). This study will be conducted in compliance with the current version of the protocol.

## Discussion

The combination of an aging Australian population and greater prevalence of CRDs in the older population (Lung Foundation Australia, 2022) means the demand for PR will only increase in the future. Thus, there is a need for research into alternative models of the provision of PR to increase accessibility to this highly effective intervention. If this model of delivery of PR by upskilling of the existing physiotherapy and exercise physiology workforce and utilization of existing healthcare infrastructure is shown to be feasible, future research will need to test its clinical effectiveness, cost-effectiveness, and wider uptake. A strength of this model of delivery of PR is that it occurs entirely within the Australian primary health care system, however, uptake by clinicians and patients and sustainability of such a model may depend on an appropriate funding pathway. Most existing PR services are provided through hospital outpatient models, where there are extensive waiting lists. If PR can be provided by private physiotherapy or AEP practices, patients will be given greater autonomy and choice on where their PR takes place. Practices often open after regular business hours, which may give people with work commitments the opportunity to undertake PR. A need for a funding scheme that allows primary care clinicians to provide PR within their practices is supported by the recent COPD Blueprint (Lung Foundation Australia, 2022). Further, promotion of multidisciplinary models of care has been flagged in the Strengthening Medicare Taskforce Report (Australian Government, 2023).

Strengths of this study include its design, being two phases to evaluate both the upskilling of physiotherapists and AEPs and delivery of PR entirely within the primary care setting. An understanding of the requirements needed to upskill primary care clinicians to provide PR in a PP setting is a gap in the current knowledge. The mixed-methods nature of this study will allow examination of the effectiveness and appropriateness of the clinician upskilling programme and the participants’ experiences.

The study has some limitations. First, there is no follow-up of patient participants past the end of the intervention period to evaluate the longer-term impacts of the intervention. Also, the study will be conducted in a single metropolitan area, meaning further research may need to be conducted in different settings for generalizability of the results. Further, the study is not powered to examine the effectiveness of PR on clinical outcomes. However, this feasibility trial aims to provide data that will lead to the design and conduct of a full-scale randomized controlled trial if the intervention is found to be feasible. The full-scale trial would provide an opportunity for investigation of the effect of the intervention on healthcare utilization and cost-effectiveness which will be important for informing policy change to support PP physiotherapists and AEPs to provide PR in primary care.

## Supporting information

Walsh et al. supplementary material Walsh et al. supplementary material

## Data Availability

The data that support the findings of this study are available from the corresponding author upon reasonable request.

## Supplementary material

The supplementary material for this article can be found at https://doi.org/10.1017/S1463423625100340.
