# PURE PRIME: Implementing pulmonary rehabilitation in primary care: a protocol for a randomized controlled feasibility trial – CORRIGENDUM

**DOI:** 10.1017/S1463423625100479

**Published:** 2025-10-01

**Authors:** Jessica A. Walsh, Zoe J. McKeough, Marita T. Dale, Jennifer A. Alison, Sarah M. Dennis

The authors regret that this article was published with two references missing from the reference list.

In Table 1, the citation that currently reads *Williams et al. 2014* is incorrect. This should have referred to: Stanojevic S, Kaminsky DA, Miller M, et al. ERS/ATS technical standard on interpretive strategies for routine lung function tests. Eur Respir J. 2022 Jul 13;60(1):2101499.

In Table 2, the three citations within the table that currently read *Zakrisson et al. 2011* should instead have referred to: Borg G. Psychophysical bases of perceived exertion. Med Sci Sports Exerc. 1982;14(5):377-381.
