# Supplementary material for: PURE PRIME: Implementing PUlmonary REhabilitation in PRIMary carE: a protocol for a randomized controlled feasibility trial
Source: Prim Health Care Res Dev. 2025 Aug 15;26:e71. doi: 10.1017/S1463423625100340 (PMC12455362; doi:10.1017/S1463423625100340)
Supplement: Walsh et al. supplementary material [file S1463423625100340sup001.pdf]

Knowledge questionnaire

Below is a case study of a COPD patient. Please read the case study and then answer the questions that follow.

If you have any questions, please contact Jessica Walsh, jwal2265@uni.sydney.edu.au or 0481127212

Case Study Overview

Patrick is a 65 year old male. He recently experienced an exacerbation which led to a hospital admission. The GP surgery received a notification of discharge from the hospital and you, as the pulmonary rehabilitation clinician, have been asked to make contact with Patrick and arrange for him to come in for an assessment.

History

Patrick has no cardiac issues, no chest pain, and a normal BP. He has osteoarthritis in hips and knees, but has not had any surgery. Patrick has no history of depression.

Smoking

Patrick did smoke for 45 years, averaging 22 cigarettes per day. He quit at aged 60 after several unsuccessful attempts.

Nutrition

Patrick has a BMI of 27.1 kg/m2, putting him in the overweight category.

Alcohol

Patrick drinks around 8 beers a week. He has 1 can on 4 days and 2 cans on a Friday and Saturday night. (375 ml can of mid strength beer = 1 standard drink).

Activity

Has not previously been referred to pulmonary rehabilitation. Mobilises independently. He is currently not exercising on a regular basis. He is breathless when walking up hills and stairs. He can manage his ADLs but has to stop and rest after a few minutes. The hardest activity for him is lifting and climbing hills and stairs.

Goals

Patrick wants to understand more about his disease. He wants to feel fitter and stronger.

Psychosocial

Patrick lives alone - his wife passed away 2 years ago. He has no family living close by.

Medication

Patrick takes Spiriva in the morning, Seretide morning and evening, and Ventolin as needed. He usually remembers to take his medication. He does not use a spacer but remembers the nurse mentioning it in hospital.

Use the case study above to answer the following questions

1) What would you do with Patrick when he first comes to pulmonary rehabilitation? Choose 4 options.

- ☐ Take a full medical history
- ☐ Explain what the pulmonary rehabilitation program involves
- ☐ Assess functional exercise capacity using the spirometer
- ☐ Assess functional exercise capacity using the six-minute walk test
- ☐ Assess quality of life using the St George's Respiratory Questionnaire

2) What are the main components of the pulmonary rehabilitation program? Choose 2 options.

- ☐ Exercise training
- ☐ Self-management education
- ☐ Blood pressure monitoring
- ☐ Modified MRC Dyspnoea Scale
- ☐ All of the above

(Note: If the option 'all of the above' is chosen, two options do not need to be chosen)

3) What would be the four main types of exercise training you would prescribe for Patrick?

- ☐ Endurance training for the lower limbs
- ☐ Yoga
- ☐ Endurance training for the upper limbs
- ☐ Strength training for the lower limbs
- ☐ Balance training
- ☐ Strength training for the upper limbs
- ☐ Pilates
- ☐ None of the above

4) What are the best two exercises you could prescribe for lower limb endurance training for Patrick?

- ☐ Crawling
- ☐ Pilates
- ☐ Walking
- ☐ Cycling
- ☐ Sit to stand exercises
- ☐ All of the above
- ☐ None of the above

(Note: If the option 'all of the above' is chosen, two options do not need to be chosen)

5) What equation would you use to determine the intensity of walking training for 20 minutes for Patrick?

- ◯ [(6MWDx10)0.8]/3
- ◯ [(6MWDx6)0.8]/3
- ◯ [(6MWDx8)0.8]/3
- ◯ [(6MWDx10)0.6]/3
- ◯ None of the above

6) What training prescription would you choose for upper limb strength training for Patrick?

- ◯ A weight that can be lifted/pushed 8 times without too much difficulty; 3 sets of 15-20 repetitions
- ◯ A weight that can be lifted/pushed 15 times without too much difficulty; 3 sets of 8-10 repetitions
- ◯ A weight that can be lifted/pushed 1 time without too much difficulty; 5 sets of 10-12 repetitions
- ◯ A weight that can be lifted/pushed 8 times without too much difficulty; 3 sets of 8-10 repetitions

7) What three pieces of exercise equipment could you use for upper limb strength training for Patrick?

- ☐ Hand weights (dumbbells)
- ☐ Theraband
- ☐ Weight machines
- ☐ Bicycle
- ☐ Arm ergometer

8) What is the evidence-based recommendation or frequency and duration of Patrick's pulmonary rehabilitation program? More than one option may be correct.

- ☐ Twice per week for 20 weeks
- ☐ Twice per week plus do home exercise for 8 weeks
- ☐ 3 times per week for 8 weeks
- ☐ Whenever he likes
- ☐ Once a week for 6 weeks

9) Considering Patrick's history, what will most likely be the limiting factors when he is exercising? Choose three options.

- ☐ Breathlessness
- ☐ Joint pain
- ☐ Leg fatigue
- ☐ Chest pain
- ☐ Headache
- ☐ Thirst

10) Considering Patrick's history and goals, what topics are important to cover in the self-management education?

- ◯ Airways and clearance techniques
- ◯ Medications and devices
- ◯ Management of breathlessness
- ◯ Understanding lung disease
- ◯ Importance of regular exercise and physical activity
- ◯ All of the above

11) When Patrick completes pulmonary rehabilitation, what should you encourage him to do? Choose two options.

- ☐ Continue to exercise at home
- ☐ Join a community exercise class e.g. Lungs in Action
- ☐ Go on a cruise and relax
- ☐ Exercise if and when he feels able to
- ☐ All of the above
